# Factors Associated with Virological Failure and Suppression after Enhanced Adherence Counselling, in Children, Adolescents and Adults on Antiretroviral Therapy for HIV in Swaziland

**DOI:** 10.1371/journal.pone.0116144

**Published:** 2015-02-19

**Authors:** Kiran Jobanputra, Lucy Anne Parker, Charles Azih, Velephi Okello, Gugu Maphalala, Bernard Kershberger, Mohammed Khogali, Johnny Lujan, Annick Antierens, Roger Teck, Tom Ellman, Rose Kosgei, Tony Reid

**Affiliations:** 1 Médecins Sans Frontières (Operational Centre Geneva), Mbabane, Swaziland; 2 Swaziland National ART Program, Ministry of Health, Mbabane, Swaziland; 3 Swaziland National Reference Laboratory, Mbabane, Swaziland; 4 Médecins Sans Frontières (Operational Research Unit), Luxembourg, Luxembourg; 6 Médecins Sans Frontières (Operational Centre Geneva), Geneva, Switzerland; 7 Médecins Sans Frontières (Southern Africa Medical Unit), Cape Town, South Africa; University of Athens, Medical School, GREECE

## Abstract

**Introduction:**

This study explores factors associated with virological detectability, and viral re-suppression after enhanced adherence counselling, in adults and children on antiretroviral therapy (ART) in Swaziland.

**Methods:**

This descriptive study used laboratory data from 7/5/2012 to 30/9/2013, which were linked with the national ART database to provide information on time on ART and CD4 count; information on enhanced adherence counselling was obtained from file review in health facilities. Multivariable logistic regression was used to explore the relationship between viral load, gender, age, time on ART, CD4 count and receiving (or not receiving) enhanced adherence counselling.

**Results:**

From 12,063 patients undergoing routine viral load monitoring, 1941 (16%) had detectable viral loads. Children were more likely to have detectable viral loads (AOR 2.6, 95%CI 1.5–4.5), as were adolescents (AOR 3.2, 95%CI 2.2–4.8), patients with last CD4<350 cells/µl (AOR 2.2, 95%CI 1.7–2.9) or WHO Stage 3/4 disease (AOR 1.3, 95%CI 1.1–1.6), and patients on ART for longer (AOR 1.1, 95%CI 1.1–1.2). At retesting, 450 (54% of those tested) showed viral re-suppression. Children were less likely to re-suppress (AOR 0.2, 95%CI 0.1–0.7), as were adolescents (AOR 0.3, 95%CI 0.2–0.8), those with initial viral load> 1000 copies/ml (AOR 0.3, 95%CI 0.1–0.7), and those with last CD4<350 cells/µl (AOR 0.4, 95%CI 0.2–0.7). Receiving (or not receiving) enhanced adherence counselling was not associated with likelihood of re-suppression.

**Conclusions:**

Children, adolescents and those with advanced disease were most likely to have high viral loads and least likely to achieve viral suppression at retesting; receiving adherence counselling was not associated with higher likelihood of viral suppression. Although the level of viral resistance was not quantified, this study suggests the need for ART treatment support that addresses the adherence problems of younger people; and to define the elements of optimal enhanced adherence support for patients of all ages with detectable viral loads.

## Introduction

In 2013 the World Health Organisation (WHO) recommended routine annual viral load (VL) monitoring for all patients on antiretroviral therapy (ART), as the most accurate available measure of effective treatment response.[1] An elevated or ‘non-suppressed’ VL (>1000 copies/ml) in a patient who has been on ART for at least six months can indicate either therapeutic failure due to antiretroviral resistance, and / or poor adherence to treatment. To distinguish between these two conditions, a patient with an elevated VL should receive adherence support followed by retesting three to six months later.[1] According to the WHO guidelines, patients whose VLs are not suppressed at retesting can be classified as having ‘virologic failure’ due to probable drug resistance, and should be switched to second line therapy.[1]

Previous studies on VL monitoring suggest a number of factors that may be associated with having an elevated VL, and subsequent virologic failure. Patients on treatment for more than one year are more likely to have virologic failure and to have resistance mutations.[2] Children are also more likely to have persistently elevated VLs despite adherence support.[3] However, children and adolescents often have challenging adherence problems, related to inadequate drug formulations, social context (e.g. lack of consistent care-giver in younger children) and psycho-developmental stage (e.g. disclosure and acceptance of diagnosis in adolescents), which may not respond to conventional forms of adherence support.[4] Young people may therefore fail to achieve viral re-suppression following adherence counselling, without necessarily having resistant virus.[5] If indeed adherence counselling is not meeting the specific adherence-related needs of children, using the WHO 2013 algorithm could result in over-estimation of virologic failure due to resistance in younger people, resulting in inappropriate switches to second line therapy.

Swaziland has the highest prevalence of Human Immunodeficiency Virus (HIV) of any country worldwide (31% amongst the 18–49 age group), and in 2012 introduced routine VL monitoring for patients on ART as a pilot programme in the Shiselweni region.[6–7] The implementation challenges of VL monitoring in this setting have been described previously, yet there have been no published analyses of predictors of virological outcomes in Swaziland.[5] Identification of predictors of virological failure is important for health programmers since it can enable better targeting of adherence support. Although previous studies in sub-Saharan Africa have addressed this question, it is difficult to generalise these findings to Swaziland which has a relatively new ART programme and has lower levels of HIV drug resistance than other countries in the region.[ 8, 9] Furthermore, around 10% of the ART cohort in Swaziland are under the age of 20, which enables a robust description of virological outcomes in younger people in a sub-Saharan setting.[7] This analysis of factors associated with viral suppression in Swaziland thus aims to identify groups at risk of having non-suppressed VL and hence virologic failure, to enable better targeting of adherence support and referral for clinician review.

## Objectives

This primary objective of this study is to explore factors associated with virological detectability, and subsequent virologic failure in adults and children on ART in Swaziland. The secondary objective is to establish whether receiving enhanced adherence counselling is associated with likelihood of viral re-suppression, amongst patients with initial detectable VL.

## Methods

### Design

This was a descriptive study using routinely collected programme data.

### Setting

Swaziland is a landlocked, lower-middle income country in Southern Africa, with a population of 1.2 million and an adult HIV prevalence of 31%.[1] The study region (Shiselweni) has a largely rural population of 210,000, with an estimated 37,000 people living with HIV, of whom 16,349 were on ART at the end of 2012.[2] ART is provided from 22 primary care clinics and three referral facilities.

In May 2012, Médecins Sans Frontières (MSF) and the Ministry of Health of Swaziland implemented routine VL monitoring in Shiselweni, with enhanced adherence counselling (EAC) for patients with detectable VL, using a generic HIV VL platform commercialised by Biocentric (Bandol, France). In Shiselweni, patients on ART now undergo annual VL testing once they have been on ART for six months (as a measure of early adherence), and annually thereafter. Patients with detectable VLs are referred to a lay-counsellor for three months’ EAC followed by retesting the following month, at which point they are referred to a doctor if their VL remains elevated. Full details of the programme and VL monitoring algorithm have been published previously.[5]

### Participants and data collection

All patients undergoing VL testing in health facilities in Shiselweni between 7/5/2012 and 30/9/2013 were included in the analysis. VL laboratory records included information on test results and dates, age, sex and health facility. Laboratory records from all patients who underwent VL monitoring in this period were linked to the national ART database (APMR) using a unique patient identifier (ART number) to provide information on time on ART, recent CD4 count, recent WHO clinical stage, ART regimen and TB co-infection status. WHO staging and recent CD4 data were only considered relevant if the CD4 or WHO clinical stage had been taken within 12 months of the VL test date. If no CD4 or VL had been recorded within the last 12 months, these variables were coded as missing.

Trained data clerks visited the health facilities to review a consecutive sample of 200 patient files of patients with detectable VLs from 1^st^ Oct 2012 to 31^st^ March 2013, and recorded the dates of the counselling sessions following a detectable VL.

### Analysis

Two separate analyses were conducted based on two binary outcome variables:

- For the analysis of predictors of viral detectability, the outcome variable was “Initial VL”, classified as “detectable” or “undetectable” according to the detection threshold of the Biocentric technique (100copies/ml).
- For the analysis of predictors of virologic failure, the outcome variable was “Follow-up VL”, classified as “re-suppressed” or “virologic failure”, with viral re-suppression defined as undetectable VL (less than 100copies/ml), or a 2 log reduction in VL, at least 60 days after an initial detectable VL.

In view of the possibility of virological blips between 100 and 1000 copies/ml, this analysis was re-run excluding the group with a first VL in this range to ascertain whether this altered the findings.

Explanatory variables used in the analysis were gender, age, type of facility (primary or secondary health care), time on ART, last WHO clinical stage, last CD4 count, treatment regimen (1^st^ or 2^nd^ line) and TB co-infection status. For the analysis of factors associated with viral re-suppression, initial VL and number of EAC sessions received were also included as explanatory variables. Time on ART was treated as a continuous variable; patients were also categorised as having been on ART for more or less than nine months, in view of the clinical relevance of distinguishing between patients undergoing VL monitoring as a test of early adherence at six months since initiating ART, and those undergoing routine annual monitoring.

Pearson’s Chi Square test was used to compare categorical variables, and the Kruskal-Wallis test was used for comparison of medians for continuous variables with asymmetric distributions. Multivariable logistic regression [reported with Adjusted Odds Ratios (AOR) with 95% Confidence Intervals (95% CI)] was performed in a backward, step-wise manner, from an initial model which included all explanatory variables with a p-value of <0.1 on bivariate analysis, until a best-fit model was found. Data entry was carried out with EpiData 3.1, and data analysis was performed using Stata/SE (StataCorp, Texas, U.S.A.) Version 12.1.

Since the study was carried out using only routine data sources, there was a substantial proportion of missing data. For each variable of interest, we assessed whether having a missing value was associated with the outcome of interest (detectability or re-suppression) and whether the missing values were disproportionately distributed among certain groups (such as in specific health facilities). Many patients with missing values came from the same few health facilities, so clustering by health facility was controlled for in the analysis. In the final model regression models, analysis was restricted to records for which all relevant variables were available. This decision was made after exploring the potential impact on the findings of including these extra records in the analysis (see S1 Table and ‘missing data’ in the discussion below).

### Ethics

This study has received ethical approval from the MSF Ethics Review Board, Geneva, Switzerland, and the Swaziland Scientific and Ethics Committee, Mbabane, Swaziland. Since this study uses only laboratory records and information from routine clinical records, patients were not asked to give written consent for study participation. To protect patient confidentiality, all records were anonymised and de-identified prior to analysis.

## Results

### Patient characteristics and exclusions

**Figure 1:**
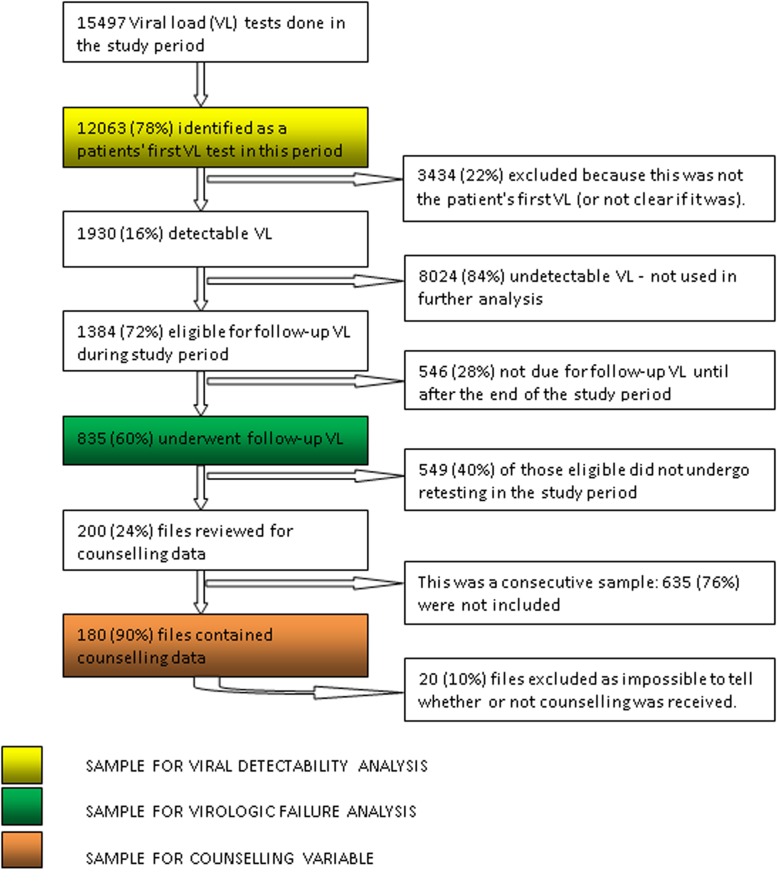
Numbers and proportions of viral load tests included and excluded at each stage of the analysis of predictors of virological outcomes, Swaziland, 2012–2013.

Fig. 1 summarises the inclusions and exclusions for the different stages of the analysis. Of 15,497 VL laboratory records from tests ordered between 7/5/2012–30/9/2013, 2151 were excluded since the patient had already had an annual test in the study period, and 1283 were excluded because they had no unique identifier recorded (thus it was unclear if the test was a routine or follow-up test). The 12,063 identifiable patients who underwent VL monitoring over this period represent 73% of the Shiselweni ART cohort of 16,349 patients, and all were included in the “Predictors of viral detectability” analysis. There was no difference in the proportion of detectable results amongst those patients that were included and those that were excluded from the study.

Many patient records had missing data for some of the explanatory variables of interest. We were able to link the laboratory records with the national ART database for 9954 (83%) of the patients. For those patient records that could not be linked, information could thus not be obtained on time on ART, CD4, WHO stage, TB status and ART regimen; these patients were more likely to have detectable VLs (see discussion and S1 Table). Amongst those records that could be linked, a number of CD4 results (5692) and WHO staging results (205) were excluded since they were obtained more than 12 months prior to the VL test. There was no difference in the proportion of detectable VLs amongst patients whose CD4 and WHO staging results were excluded, and the rest of the cohort.

Characteristics of patients at time of VL testing are shown in Tables 1 and 2, and the data is provided in S1 and S2 Datasets (although unique patient identifiers have been removed). From the full sample of 12,063 patients, 4220 (35%) were male and 1,168 (10%) were under the age of 20. For patients with available data, median time on ART was 2.7 years; and 66 (0.7%) were on second line therapy. Of those patients with CD4 and WHO staging information recorded in the last 12 months, 1421 (45%) had a CD4<350 cells/µl, and 2682 (28%) were WHO stage 3 or 4.

**Table 1:** Factors associated with detectable viral load (viral load >100 copies/ml) in patients on antiretroviral therapy for more than six months in Swaziland, 2012–2013.

	**Total n**	**Detectable n (%)**	**Odds Ratio OR (95% CI)**	**Adj Odds Ratio^1^ OR (95% CI)**
**Gender**				
Male	4220 (100%)	742 (18)	1.2 (1.1–1.3)	
Female	7824 (100%)	1196 (15)	1	
**Age group**				
<10yrs (children)	580 (100%)	170 (29)	2.5 (2.1–3.0)	2.6 (1.5–4.5)
10–19yrs (adolescents)	588 (100%)	207 (35)	3.2 (2.7–3.9)	3.2 (2.2–4.8)
20+ (adults)	10808 (100%)	1553 (14)	1	1
**Type of facility**				
Secondary facility	6267 (100%)	938 (15)	1.2 (1.1–1.3)	
Primary care clinic	5796 (100%)	1003 (17)	1	
**Time on ART** ^2^				
Median time in years(IQR)	2.7 (1.4–4.4)	2.8(1.5–4.6)^3^	1.0 (1.0–1.1)	1.1 (1.1–1.2)
< 9 months (early adherence)	922	130 (14)	0.9 (0.7–1.1)	
> 9 months (routine annual)	8985	1377(15)	1	
Unknown^5^	2156	434 (20)		
**Last WHO** ^4^ **clinical stage**				
1 or 2	7025	1024 (15)	1	1
3 or 4	2682	470 (18)	1.2 (1.1–1.4)	1.3 (1.1–1.6)
No WHO stage in last 12 m^5^	2356	447 (19)		
**Last CD4 count**				
<350 cells/µl	1421	296 (21)	1.8 (1.5–2.2)	2.2 (1.7–2.9)
350+ cells/µl	1741	218 (13)	1	1
No CD4 in last 12 m^5^	8901	1427 (16)		
**TB co-infection status**				
Current TB infection	117	22 (19)	1.3 (0.8–2.2)	
No current co-infection	5354	785 (15)	1	
TB status unknown^5^	6590	1134(17)		
**ART** ^2^ **regimen**				
1st line	9818	1516(15)	1	
2nd line	66	11 (17)	1.1 (0.6–2.2)	
Regimen unknown^5^	2181	414 (19)		

^1^ The adjusted ORs are those from the final model, and include control for clustering by health facility.

^2^ART = antiretroviral therapy.

^3^ Median time on ART in undetectable group was 2.7 years (95% CI 1.4–4.4).

^4^ WHO = World Health Organisation.

^5^ ‘Unknown’ categories are included when >1% of values are missing. Having a missing value for time on ART, WHO Clinical Stage, last C4 count, TB co-infection status or ART regimen was associated with increased likelihood of detectable VL. These patients were excluded from the final regression model (see methods and S1 Table).

**Table 2:** Factors associated with viral re-suppression (viral load<100 copies/ml, or 2 log reduction) after planned adherence counselling in patients with detectable viral load on antiretroviral therapy in Swaziland, 2012–2013.

	**Total n**	**Re-suppressed n (%)**	**Odds Ratio OR (95% CI)**	**Adj. Odds Ratio^1^ AOR (95% CI)**
**Gender**				
Male	327	170 (52)	0.9 (0.7–1.2)	
Female	507	280 (55)	1	
**Age group**				
<10yrs (children)	72	27 (38)	0.4 (0.3–0.7)	0.2 (0.1–0.7)
10–19yrs (adolescents)	98	33 (34)	0.4 (0.2–0.6)	0.3 (0.2–0.8)
20+ (adults)	661	389 (59)	1	1
**First viral load (copies/ml)**				
101–1000	170	124 (73)	1	1
1001–5000	176	92 (52)	0.4 (0.3–0.6)	0.3 (0.1–0.5)
5001–50000	291	132 (45)	0.3 (0.2–0.5)	0.3 (0.1–0.7)
over 50,000	198	103 (52)	0.4 (0.3–0.6)	0.4 (0.2–1.1)
**Type of facility**				
Secondary facility	381	214 (56)	1.2 (0.9–1.5)	
Primary care clinic	454	237 (52)	1	
**Time on ART** ^2^				
Median time in years (IQR)	2.9 (1.6–4.8)	2.8 (1.5–4.6)^3^	0.9 (0.9–1.0)	
< 9 months (early adherence) tests)	38	21 (55)	1.0 (0.5–1.9)	
> 9 months (routine annual)	674	374 (56)	1	
Unknown time on ART^5^	123	56 (46)		
**Last WHO** ^4^ **clinical stage**				
1 or 2	472	263 (56)	1	
3 or 4	236	131 (56)	1.0 (0.7–1.4)	
No WHO stage in last 12 m^5^	127	57 (45)		
**Last CD4 count**				
<350 cells/µl	146	71 (49)	0.5 (0.3–0.8)	0.4 (0.2–0.7)
350+ cells/µl	113	75 (66)	1	1
No CD4 in last 12m^5^	576	305 (53)		
**TB co-infection status**				
Current TB infection	7	4 (57)	1.2 (0.3–5.4)	
No current co-infection	393	208 (53)	1	
TB status unknown^5^	435	239 (55)		
**ART** ^2^ **regimen**				
1st line	707	393 (56)	1	
2nd line	5	3 (60)	1.2 (0.2–7.2)	
Unknown regimen^5^	123	55 (45)		
**Enhanced adherence counselling**				
No counselling	35	22 (63)	1	
1–3 counselling sessions	145	70 (48)	0.5 (0.2–1.1)	
No information collected^5^	655	359 (55)		

^1^ The adjusted ORs are those from the final model, and include control for clustering by health facility.

^2^ART = antiretroviral therapy.

^3^ Median time on ART in undetectable group was 3.2 years (95% CI 18–4.9).

^4^ WHO = World Health Organisation.

^5^ ‘Unknown’ categories are included when >1% of values are missing. On regression analysis, no association was seen between likelihood of re-suppression and having unknown missing value for time on ART, WHO Clinical Stage, last C4 count, TB co-infection status or ART regimen.

### Factors associated with having a detectable VL

On bivariate analysis, males, patients under 20 yrs old, those with WHO stages 3 or 4 disease or advanced immuno-suppression (CD4<350 cells/µl), and those managed in primary rather than secondary care locations, were more likely to have detectable VL (p<0.001 in all cases). Patients with detectable VL tended to have been on ART for longer than patients with undetectable VL, although this was not statistically significant (p = 0.08). ART regimen and TB co-infection status were not associated with virological detectability. Patients undergoing VL testing for early adherence (at six months) were no more likely to have a detectable VL than patients attending for routine annual monitoring.

In the final adjusted model, the key predictors of detectability were: being an adolescent or a child, longer time on ART, WHO Clinical Stages 3 and 4, and recent CD4<350 cells/µl. Crude and Adjusted Odds Ratios are shown in Table 1.

### Factors associated with virologic failure and re-suppression

Of the original 12,063 patients, 1,930 (16%) had a detectable VL, of whom 835 had a follow-up VL during the study period; this represents approximately 60% of those eligible for follow-up testing during the study period, on the basis of the timing of their first test. The median time to follow-up was four months. All 835 patients with a follow-up VL test were included in the “predictors of virologic failure” analysis, of whom 451 (54%) showed viral suppression at re-testing. Again, not all patients had complete data for all explanatory variables of interest, but no patient was excluded on this basis.

The review of 200 patient files to obtain information on EAC yielded 180 files with information recorded: 35 (19%) had received no EAC; 145 (81%) had received at least one session of counselling, and 36 (20%) had received all three sessions. In 20 files it was unclear whether the patient had received EAC or not. There was no difference in the rates of re-suppression among the sample of 180 patients for whom information on EAC was collected, and the rest of the cohort.

On bivariate analysis, patients younger than 20, those with VL >1000 at initial test, and those with CD4 count <350 cells/µl were more likely to show virologic failure at retesting (p<0.01 in each case). Gender, WHO clinical stage, TB co-infection status, time on ART, type of facility and ART regimen were not associated with likelihood of virologic failure. Patients who had a detectable VL at their early adherence test (at six months) appeared as likely to re-suppress as those who were detectable at routine annual monitoring. There was no difference in re-suppression rates between those who underwent EAC and those who did not. Restricting analysis to patients with a first detectable VL of over 1000 had no effect on these findings.

In the final multivariate regression model, the factors associated with virologic failure were: being a child or an adolescent, having an initial VL between 1000 and 50,000 copies/ml and recent CD4 <350 cells/µl. Crude and Adjusted Odds Ratios are shown in Table 2.

## Discussion

This programmatic study demonstrated that children and adolescents on ART were more likely to have raised VLs, and less likely to achieve viral suppression at retesting when compared with the adult cohort. Receiving EAC did not appear to increase the likelihood of viral suppression, relative to receiving a detectable VL result from the health worker.

Overall, 84% of patients in the cohort had an undetectable VL, and of those with detectable VLs, 54% resuppressed following planned adherence counselling. Likelihood of viral detectability increased with time on ART and advanced disease; virologic failure was more likely in advanced disease (as indicated by CD4 cell count). This is consistent with other studies, and suggests that these groups are more likely to have resistance mutations.[8, 10]

### Children and adolescents

Children and adolescents were significantly more likely to have a detectable VL, and less likely to re-suppress at retesting. This is consistent with the limited available evidence on VL monitoring in children, and evokes the specific barriers to adherence faced by children and adolescents, that are unlikely to be addressed successfully through counselling.[4, 11] Due to the limited availability of paediatric fixed-dose ARV formulations, children in Swaziland are often required to take a number of tablets each day, which may well contribute to this poorer adherence. Furthermore, treatment support models based primarily on counselling the caregiver may be poorly adapted to the needs of younger people. In contrast, programmes which make use of social workers, peer support and training on paediatric disclosure have reported better virological outcomes in children.[12] Since levels of paediatric drug resistance in Swaziland are relatively low [9] care must be taken to exclude ongoing adherence problems when applying the WHO 2013 algorithm to children and adolescents, to avoid inappropriate changes to second line therapy. Where feasible, more regular VL measurements and use of genotyping should be considered in these cases.

### Enhanced adherence counselling

In our study patients with raised VLs who received EAC by lay counsellors were no more likely to have a subsequent suppressed VL than those who did not receive counselling. Although factors such as age and sex were controlled for in the analysis, there may be other factors that could explain why patients who did not undergo counselling tended also to suppress their VL at re-testing. For example, these patients may be more empowered (or employed) and may have chosen not to attend counselling, instead managing to improve their adherence autonomously; likewise the health worker may have made more effort to persuade those most in need of counselling to attend counselling, which may explain the poorer than expected virologic outcomes in the counselling group Secondly, the lack of apparent difference between the counselling and non-counselling groups may reflect a weakness in counselling training and supervision. Thirdly, whilst the WHO recommends an adherence intervention for patients with detectable VL, it does not specify what form that intervention should take. We must recognise that the act (by the health worker) of explaining to a patient that their VL is detectable constitutes an intervention in itself; indeed, we can assume that all retested patients were notified of their initial result, even if they did not subsequently receive counselling. If it is this intervention that results in re-suppression, then efforts must focus on improving ‘treatment literacy’ amongst patients on ART, including awareness of the role and importance of VL testing. Finally, viral resistance was not measured in this study, and thus the possibility that rates of resistance were higher in the counselling group cannot be excluded.

### Virologic ‘blips’

Patients with an initial low detectable VL (<1000copies/ml) were more likely to resuppress; this may reflect a ‘return to mean’ phenomenon, or virologic ‘blips’, rather than indications of poor adherence or resistance.[13] Raising the threshold for EAC and retesting to 1000 copies/ml could avoid unnecessary use of counselling and laboratory resources in this group. However, this must be balanced with the evidence that chronic low-level viraemia (100–1000copies/ml) is associated with poorer health outcomes.[14]

### Missing data

Our final database had a substantial amount of missing data, predominantly where we were unable to link the VL laboratory records with the national ART database. These patients appeared more likely to have detectable VLs. It is possible that they started ART prior to the implementation of the electronic register (which explains why we could not link their laboratory records), and have thus been on ART for longer, with consequent higher likelihood of treatment failure due to acquired resistance. If this is the case, our analysis may underestimate the effect of time on ART on likelihood of viral detectability. However, we also noticed that many of these patients came from the same few health facilities, which could suggest that these facilities are delivering poorer quality of care. We controlled for clustering by health facility in order to minimise the impact this would have on our estimations.

### Strengths and limitations

This study has several strengths: the sample was relatively large and included a large number of children, with good overall testing coverage of the ART cohort. For the planned adherence counselling, we verified if counselling was actually received, which has not been reported in other similar studies to our knowledge. STROBE guidelines were followed in the reporting of the study.[15]

However, since the study uses routine programme data, conclusions are limited by issues of data quality and missing values. Where possible we have controlled for this in the analysis. For the re-suppression analysis, 40% of the ‘detectable’ patients failed to undergo retesting during the study period; it is possible that this 40% are more likely to remain detectable than the 60% who successfully underwent retesting. Since the Swaziland VL programme is new, this analysis included patients having their first VL test at six months, as well as patients having a first test after many years on ART; once the programme is well established, it will be possible to compare these early adherence tests results with the results from patients undergoing annual monitoring who are known to have been virally suppressed at previous VL monitoring. Due to operational constraints we only collected data on counselling for a sub-sample of the detectable cohort; but since rates of re-suppression in this sub-sample were similar to those in the whole detectable cohort, this is unlikely to affect the overall results. Finally, this study does not look at clinical outcomes or make use of viral genotyping of patients with virologic failure to confirm the presence of resistance mutations. Whilst this would certainly be of programmatic interest, it would require a prospective cohort study and is outside the scope of this descriptive analysis.

## Conclusions and Recommendations

VL monitoring has the potential to improve ART treatment support, but clinicians should take into account the patient’s age and disease status when interpreting the VL results, and should make efforts to exclude ongoing adherence problems before considering second line therapy. This is especially the case in young people, as the adherence barriers they face may not be amenable to adult-oriented counselling; tailored treatment support with more regular VL monitoring may be helpful in this group.

For patients with elevated VLs, the act of communicating (and explaining) their result, which can be seen as improving ‘treatment literacy’, may be a key element of any adherence intervention. Further operational research should aim to define the other components of effective adherence support for patients with detectable VL.

## Supporting Information

S1 Table Factors associated with having a detectable viral load or viral re-suppression, with inclusion of subgroups of patients with missing data, in patients on antiretroviral therapy in Swaziland, 2012–13. (DOC)

S1 Dataset Database of viral load results from routine monitoring, Swaziland 2012–2013. (XLS)

S2 Dataset Follow-up database of patients with detectable viral loads, Swaziland 2012–2013. (XLS)
