# Supplementary material for: Factors Associated with Virological Failure and Suppression after Enhanced Adherence Counselling, in Children, Adolescents and Adults on Antiretroviral Therapy for HIV in Swaziland
Source: PLoS One. 2015 Feb 19;10(2):e0116144. doi: 10.1371/journal.pone.0116144 (PMC4335028; doi:10.1371/journal.pone.0116144)
Supplement: S1 Table — (DOC) [file pone.0116144.s001.doc]

**Table S1: Factors associated with having a detectable viral load or viral re-suppression (viral load<100 copies/ml or 2 log drop), showing associations with having missing values for these variables**^1^**, in patients on antiretroviral therapy in Swaziland, 2012-13**

|  |  | | | **Total tested** | | **Detectable** | | | **Odds Ratio** |  | **Total re-tested** | **Re-suppressed** | **Odds Ratio** |
| --- | --- | --- | --- | --- | --- | --- | --- | --- | --- | --- | --- | --- | --- |
|  |  | | | **n** | | **n (%)** | | | **OR (95% CI)** |  | **n** | **n (%)** | **OR (95% CI)** |
|  | |  |  | |  | | |  | | | | | |
| **Time on ART**^2^ | | | | | | | | | | | | | |
|  | Median time in years (IQR) | | | 2.7 (1.4-4.4)  (1.4 - 4.4) | | | 2.8(1.5-4.6) ^1^ | | 1.0 (1.0-1.1)  0.08^3^ |  | 2.9 (1.6-4.8)  (1.6 - 4.8) | 2.8 (1.5-4.6)  (1.5 - 4.6) | 0.9 (0.9-1.0) |
|  | < 9 months (early adherence) tests) | | | 922  (100%) | | | 130 (14) | | 0.9 (0.7-1.1)  0.32 |  | 38  (100%) | 21 (55)  (55.3%) | 1.0 (0.5-1.9)  0.98 |
|  | > 9 months (routine annual) | | | 8985  (100%) | | | 1377(15) | | 1 |  | 674  (100%) | 374 (56)  (55.5%) | 1 |
|  | Unknown time on ART | | | 2156  (100%) | | | 434 (20) | | 1.4 (1.2-1.6)^3^ |  | 123 | 56 (46) | 0.7 (0.5-1.0) |
|  | |  |  | |  | | |  | | | | | |
| **Last WHO**^4^ **clinical stage** | | | | | | | | | | | | | |
|  | 1 or 2 | | | 7025  (100%) | | 1024 (15) | | | 1  <0.001 |  | 472  (100%) | 263 (56)  (55.7%) | 1 |
|  | 3 or 4 | | | 2682  (100%) | | 470 (18) | | | 1.2 (1.1-1.4) |  | 236  (100%) | 131 (56)  (55.5%) | 1.0 (0.7-1.4)  (44.5%) |
|  | No WHO stage in last 12 m | | | 2356  (100%) | | 447 (19) | | | 1.4 (1.2-1.6)^3^ |  | 127 | 57 (45) | 0.6 (0.4-1.0) |
|  | |  |  | |  | | |  | | | | | |
| **Last CD4 count** | | | | | | | | | | | | | |
|  | <350 cells/µl | | | 1421  (100%) | | 296 (21) | | | 1.8 (1.5-2.2)  <0.001 |  | 146  (100%) | 71 (49)  (48.6%) | 0.5 (0.3-0.8) |
|  | 350+ cells/µl | | | 1741  (100%) | | 218 (13) | | | 1 |  | 113  (100%) | 75 (66)  (66.4%) | 1  (33.6%) |
|  | No CD4 in last 12m | | | 8901  (100%) | | 1427 (16) | | | 1.3 (1.1-1.6)^3^ |  | 576 | 305 (53) | 0.6 (0.4-0.9) |
|  | |  |  | |  | | |  | | | | | |
| **TB co-infection status** | | | | | | | | | | | | | |
|  | Current TB infection | | | 117  (100%) | | 22 (19) | | | 1.3 (0.8-2.2)  0.21 |  | 7  (100%) | 4 (57)  (52.9%) | 1.2 (0.3-5.4)  (47.1%)  0.83 |
|  | No current co-infection | | | 5354  (100%) | | 785 (15) | | | 1 |  | 393  (100%) | 208 (53)  (57.1%) | 1 |
|  | TB status unknown | | | 6590 | | 1134(17) | | | 1.2 (1.1-1.3)^3^ |  | 435 | 239 (55)  (54.9%) | 1.1 (0.8-1.4)  (45.1%) |
|  | |  |  | |  | | |  | | | | | |
| **ART regimen** | | | | | | | | | | | | | |
|  | 1st line | | | 9818  (100%) | | 1516(15) | | | 1  0.70 |  | 707  (100%) | 393 (56)  (62.9%) | 1 |
|  | 2nd line | | | 66  (100%) | | 11 (17) | | | 1.1 (0.6-2.2) |  | 5  (100%) | 3 (60)  (60.0%) | 1.2 (0.2-7.2)  (40.0%) |
|  | Unknown regimen | | | 2181  (100%) | | 414 (19) | | | 1.3 (1.1-1.4)^3^ |  | 123 | 55 (45) | 0.6 (0.4-0.9) |
|  | |  |  | |  | | |  | | | | | |
| **Enhanced adherence counselling** | | | | | | | | | | | | | |
|  | No counselling | | |  | |  | | |  |  | 35  (100%) | 22 (63)  (62.9%) | 1  0.18 |
|  | 1-3 counselling sessions | | |  | |  | | |  |  | 145  (100%) | 70 (48)  (51.4%) | 0.5 (0.2-1.1) |
|  | No information collected^2^ | | |  | |  | | |  |  | 655  (100%) | 359 (55)  (54.8%) | 0.7 (0.4-1.5)  (45.2%) |
|  |  | | |  | |  | | |  |  |  |  |  |

^1^ Explanatory variables with a substantial proportion (>1%) of missing values are presented here. ORs of having a detectable viral load, (and viral re-suppression) for the missing value group are shown.

^2^ ART = antiretroviral therapy

^3^ These associations persist after controlling for confounding factors (see ‘Missing values’ in the discussion section). Records with missing values were omitted from the final regression model, after exploring the effect on the apparent associations of different assumptions about the missing values. For example:

- If we assume that all those with missing time on ART had been on ART for more than 9 months, we observe that individuals on ART for longer are more likely to have a detectable VL than those on ART for less than 9 months. The analysis restricted to individuals with available data came to the same conclusion, so we chose to present this data and discuss the fact that this may underestimate the true effect of time on ART on likelihood of viral detectability.
- Similarly, if we assume that those with missing WHO Clinical stage were more likely to be stage III or IV, (or that those with missing CD4 had CD4<350), the association we reported would remain, but with a slightly lower effect size.
- For TB co-infection status or ART regimen our assumptions could be that none of those with missing values had TB and that all were on a first line ART regimen. If we use this assumption, we observe that neither variable is associated with viral detectability. This is the same conclusion as the one presented, in which the analysis was restricted to individuals with available data.

^4^ WHO = World Health Organisation
